# Retraction Note: Butein inhibits metastatic behavior in mouse melanoma cells through VEGF expression and translation-dependent signaling pathway regulation

**DOI:** 10.1186/s12906-023-04010-y

**Published:** 2023-05-29

**Authors:** Yu-Wei Lai, Shih-Wei Wang, Chien-Hsin Chang, Shih-Chia Liu, Yu-Jen Chen, Chih-Wen Chi, Li-Pin Chiu, Shiou-Sheng Chen, Allen W. Chiu, Ching-Hu Chung

**Affiliations:** 1 Department of Urology, National Yang-Ming University School of Medicine, Taipei, Taiwan; 2 Division of Urology, Taipei City Hospital Renai Branch, Taipei, Taiwan; 3 Department of Medicine, Mackay Medical College, No. 46, Sec. 3, Jhong-Jheng Rd., Sanzhi Dist, Taipei, Taiwan; 4 Institute of Pharmacology, College of Medicine, National Taiwan University, Taipei, Taiwan; 5 Department of Orthopaedics, Mackay Memorial Hospital, Taipei, Taiwan; 6 Department of Medical Research, Mackay Memorial Hospital, Taipei, Taiwan; 7 Department of Radiation Oncology, Mackay Memorial Hospital, Taipei, Taiwan

**Retraction Note: BMC Complement Med Ther 15, 445 (2015)**

10.1186/s12906-015-0970-3

The Editor has retracted this article because the authors have not been able to provide evidence that they obtained appropriate approval by an animal ethics committee for this study. In addition, Fig. 4A (panel Total-ERK) appears to overlap with Fig. 4A (panel ERK 1/2) of [1]. Ching-Hu Chung disagrees with this retraction. None of the other authors has responded to correspondence from the Editor about this retraction.
